# Comparative Pharmacovigilance Analysis of Approved and Repurposed Antivirals for COVID-19: Insights from EudraVigilance Data

**DOI:** 10.3390/biomedicines13061387

**Published:** 2025-06-05

**Authors:** Paul Andrei Negru, Delia Mirela Tit, Andrei Flavius Radu, Gabriela Bungau, Raluca Anca Corb Aron, Ruxandra Cristina Marin

**Affiliations:** 1 Doctoral School of Biological and Biomedical Sciences, University of Oradea, 410087 Oradea, Romania; negru.paulandrei@student.uoradea.ro (P.A.N.); andreiflavius.radu@uoradea.ro (A.F.R.); marin.ruxandracristina@student.uoradea.ro (R.C.M.); 2 Department of Preclinical Disciplines, Faculty of Medicine and Pharmacy, University of Oradea, 410073 Oradea, Romania; raluca.aron@didactic.uoradea.ro; 3 Department of Pharmacy, Faculty of Medicine and Pharmacy, University of Oradea, 410028 Oradea, Romania; 4 Department of Psycho-Neurosciences and Recovery, Faculty of Medicine and Pharmacy, University of Oradea, 410073 Oradea, Romania

**Keywords:** COVID-19, antivirals, EudraVigilance, pharmacovigilance, system organ class, individual case safety reports

## Abstract

**Background/Objectives**: During the COVID-19 pandemic, several antivirals were approved or repurposed, but their safety profiles have not been fully compared. Pharmacovigilance data help clarify how these drugs perform in real-world use. **Methods**: This study performed a comparative pharmacovigilance analysis of eight antivirals used or tested during the COVID-19 pandemic, based on individual case safety reports (ICSRs) retrieved from the EudraVigilance database, reported up to 9 February 2025 and extracted from the official platform on 12 February 2025. Adverse reactions were assessed by system organ class (SOC), demographic patterns, and seriousness, and disproportionality analysis (reporting odds ratio (ROR)) was conducted to identify potential safety signals. **Results**: A total of 64,776 ICSRs were analyzed. Among approved antivirals, nirmatrelvir/ritonavir (NTV/r) accounted for 13.4% (*n* = 8693) of reports, while remdesivir (RDV) represented 6.3% (*n* = 4105). Repurposed antivirals such as ribavirin and lopinavir/ritonavir dominated the dataset, together making up over 80% (*n* = 51,978) of all reports. RDV was associated with a high proportion of serious adverse events (84%, *n* = 3448), and showed consistent ROR signals in hepatobiliary, renal, cardiac, and general disorders, with values exceeding 2 in several comparisons. NTV/r displayed a milder overall profile, but with positive RORs for psychiatric disorders, gastrointestinal disorders, and product-related issues. The most affected SOCs across all drugs included general disorders (31.6%, *n* = 20,493), gastrointestinal (19.5%, *n* = 12,625), nervous system (17.8%, *n* = 11,511), and investigations (20.4%, *n* = 13,219). Demographic analysis showed that most events occurred in adults aged 18–64, with RDV more often reported in elderly patients and NTV/r more frequently associated with reports from female patients and non-healthcare reporters. **Conclusions**: This study highlights distinct pharmacovigilance profiles of COVID-19 antivirals and supports the role of real-world data in guiding safer therapeutic choices.

## 1. Introduction

Since its emergence in late 2019, coronavirus disease 2019 (COVID-19), caused by the severe acute respiratory syndrome coronavirus 2 (SARS-CoV-2), has presented a new global health threat [1]. To date, nearly 7 million individuals worldwide have died from the disease and 778 million cases of illness have been reported to the World Health Organization (WHO), although the true health burden of the pandemic has yet to be fully quantified [2].

However, improvements in the scientific study of the prevention and treatment of SARS-CoV-2 infection were made in a very short time. Although the quick discovery and deployment of vaccinations has greatly altered the pandemic’s course, the continued evolution of virus strains and the variability of individual immune responses underscore the need of treatment alternatives. Widespread implementation of SARS-CoV-2 vaccination programs, alongside advancements in standard therapeutic care, has led to a substantial reduction in COVID-19–related morbidity and mortality [3]. A wide range of pharmacological treatments, including antiviral medicines, monoclonal antibodies, and anti-inflammatory chemicals, have been studied with varied degrees of effectiveness. As additional data from clinical trials and real-world situations become available, the scientific community gains a more nuanced knowledge of therapy effectiveness across disease stages and patient demographics [4].

COVID-19 therapy is highly dependent on the disease’s severity, patient risk factors, and stage of infection. Management techniques have changed dramatically since the pandemic began, and now include a combination of supportive care, antiviral medicines, immunomodulators, and preventative approaches [5]. Antiviral medications can be delivered early after diagnosis in individuals at risk of developing severe disease, as well as drugs to enhance prognosis if severe disease has established [6]. While several drugs such as remdesivir (RDV) and nirmatrelvir/ritonavir (NTV/r) received formal approval, a wide range of other antivirals, either used off-label or repurposed from previous indications (e.g., human immunodeficiency virus (HIV) or influenza), were investigated as potential treatments. Drug repurposing is a less expensive and faster way to produce new medications. Consequently, drugs such as RDV, favipiravir (FVP), that received approval in Japan in 2014 for the management of pandemic influenza virus infections [7], umifenovir, lopinavir (LPV), ritonavir (RTV), and darunavir (DRV) were used in clinical settings to treat COVID-19. Some of these medications, such as LPV, RDV, and DRV derivates, indicated inhibition of SARS-CoV-2 multiplication in vitro on cell cultures [8,9]. RDV, molnupiravir, and nirmatrelvir (NTV) and RTV, function by inhibiting viral replication pathways, hence lowering viral load and improving therapeutic outcomes.

RDV, a nucleotide analog prodrug, targets the viral RNA-dependent RNA polymerase (RdRp), preventing SARS-CoV-2 replication [10]. RDV showed strong antiviral efficacy against SARS-CoV-2 in primary human airway epithelial cultures and human lung cells. It inhibits SARS-CoV-2 replication in a dose-dependent manner, with a half-maximal effective concentration [11]. RDV was the first antiviral to acquire Emergency Use Authorisation from the US Food and Drug Administration (FDA) in May 2020, and it later received full approval for the treatment of hospitalized patients with confirmed COVID-19 who require supplementary oxygen [12,13]. Given its broad systemic use, continued monitoring of remdesivir’s safety profile remains essential, not only to assess general tolerability, but also to better understand potential organ-specific risks, particularly those affecting the cardiovascular system. Clinical reports have linked remdesivir to events such as bradycardia, hypotension, QT interval prolongation, and atrial fibrillation, with increased susceptibility observed in patients with pre-existing heart conditions [14]. The relevance of pharmacovigilance databases in identifying cardiovascular and cerebrovascular risks has been increasingly demonstrated. A recent WHO pharmacovigilance study identified disproportionally higher reporting of myocardial infarction, stroke, hypertension and other cardiovascular events following intravitreal anti-vascular endothelial growth factor therapy [15].

NTV/r, an oral antiviral, provides a more accessible outpatient therapy alternative than RDV, which requires intravenous administration. NTV/r, an oral combination therapy, is made up of two components: NTV, a SARS-CoV-2 major protease (Mpro) inhibitor that blocks viral polyprotein cleavage, and ritonavir, a pharmacokinetic enhancer that increases Nirmatrelvir’s plasma levels by inhibiting cytochrome P450 enzymes. NTV/r was approved for emergency use in December 2021 and is suggested for those with mild to moderate COVID-19 who are at high risk of advancing to severe illness. Clinical studies show that when delivered within five days after symptom start, NTV/r lowers the risk of hospitalization or death by up to 89% compared to placebo [16]. NTV targets the SARS-CoV-2 main protease, a critical enzyme for viral replication, and has been demonstrated to minimize hospitalization and death in high-risk patients when administered early in the infection cycle [17].

Beyond RDV and NTV/r, several other antiviral agents, whether approved, repurposed, or used off-label, have been explored for the treatment of COVID-19, particularly in the early phases of the pandemic when therapeutic options were limited. Table 1 provides an overview of some of these antiviral drugs, summarizing their original indications, modes of deployment during the pandemic, and known adverse effects.

Many of these drugs saw widespread use early in the pandemic, often before targeted COVID-19 treatments were available. While most have since returned to their original uses, their large-scale, often off-label use raised valid safety concerns. Unlike controlled clinical trials, real-world safety data, gathered through systems such as EudraVigilance, reflect how these drugs perform across diverse populations, comorbidities, and treatment contexts. These spontaneous reports help identify potential safety issues that might go unnoticed in trials, particularly when drugs are used off-label or outside formal protocols.

EudraVigilance is a centralized European database managed by the European Medicines Agency (EMA) for the collection, management, and analysis of suspected adverse reactions to medicines authorized in the European Economic Area (EEA). It supports pharmacovigilance activities by enabling early detection of safety signals and risk assessment across Europe. The database contains individual case safety reports (ICSRs) submitted by healthcare professionals, patients, and pharmaceutical companies, and is accessible to regulatory authorities, healthcare providers, and, in part, the public through the adverse drug reaction portal [42].

The present study aims to compare the safety profiles of the main antivirals used in the context of COVID-19, including both medications formally approved for this indication (i.e., RDV and NTV/r) and others that were repurposed or used off-label during various stages of the pandemic. Using data from the EudraVigilance database, the study evaluates the frequency and seriousness of reported adverse events, the most affected organ systems, and potential safety signals (ROR). The ultimate goal was to support more informed treatment decisions and strengthen real-world safety monitoring of antiviral therapies.

## 2. Materials and Methods

### 2.1. Study Design and Data Collection

This retrospective, descriptive study is based on pharmacovigilance data retrieved from individual case safety reports (ICSRs), submitted to the EudraVigilance database, reported up to 9 February 2025 and extracted from the official platform, accessed on 12 February 2025 [43]. The analysis included only those antiviral agents for which EV data were available at the time of extraction.

Three categories of antivirals were included in this study, based on their approval status and clinical use during the COVID-19 pandemic, and depending on the availability of data in the EudraVigilance (EV) database. The first category included officially authorized treatments for COVID-19, namely remdesivir (RDV) and nirmatrelvir/ritonavir (NTV/r), which served as the main reference antivirals. The second category contained favipiravir (FVP). Although not formally approved for COVID-19, FVP was used off-label in several countries and was retained in the analysis due to the presence of safety reports in the EudraVigilance database. The third category consisted of other repurposed antivirals that were studied during the pandemic but were not adopted in standard treatment protocols. These included lopinavir/ritonavir (LPV/r), ribavirin (RBV), nelfinavir (NFV), atazanavir (ATV), and darunavir (DRV).

All reported adverse events were coded using the Medical Dictionary for Regulatory Activities (MedDRA), a clinically validated international terminology developed by the International Council for Harmonization (ICH) and widely used by regulatory authorities. MedDRA is a hierarchical system that organizes medical terms based on anatomical, pathological, physiological, etiological, or functional criteria. At its highest level, adverse reactions are classified into 27 System Organ Classes (SOCs), which encompass multiple high-level group terms and associated preferred terms [44]. Given the structure’s flexibility, particularly at broader levels, some variability in coding practices may occur. For this reason, results aggregated at the SOC level should be interpreted with appropriate caution.

For each antiviral, the number of ICSRs was extracted and grouped by SOC category. In addition to the overall number of reports, the seriousness of each case was also considered. Reports were classified as either serious (e.g., resulting in hospitalization, disability, life-threatening events, or death) or non-serious, based on the criteria established within the EudraVigilance reporting system. Consistent with the ICH E2A guidelines, congenital anomalies or birth defects are classified as serious adverse events, regardless of clinical severity or outcome [45]. The analysis did not require ethical approval since the data used is anonymous, and patient identification is not possible through the information available in the EV database.

### 2.2. Statistical Analysis

The statistical analysis was performed using OpenEpi software v3.01 (Atlanta, GA, USA) [46] and supported by Microsoft Excel and JASP for data visualization and exploratory comparisons. The descriptive analysis included patient demographic characteristics, according to European Medicines Agency (EMA) recommendations. Data were grouped by age categories (0–1 month, 2 months–2 years, 3–11 years, 12–17 years, 18–64 years, 65–85 years, over 85 years), gender (female, male, unspecified), geographical origin (European Economic Area—EEA, non-EEA, unspecified), and reporter category. Reported categories were classified as healthcare professionals (e.g., physicians, nurses, pharmacists), non-healthcare professionals (mainly patients or caregivers), or unspecified. The cases were classified according to their severity (serious, non-serious, unspecified) for identifying the trends specific to each antiviral. Since individual ICSRs may contain multiple adverse reactions, the average number of ADRs per report was calculated to reflect the overall reporting burden.

The analysis methodically compares the safety profiles of the selected antivirals, with the following parameters being considered: the total number of ICSRs reported in relation to each drug, the classification of suspected adverse reactions (ADRs) according to the affected SOC, and disproportionality analysis in order to highlight any potential safety signals [47]. Disproportionality analysis was performed using the reporting odds ratio (ROR) method, with OpenEpi (version 3.01) used to compute ROR values and their 95% confidence intervals (CI). Approved antivirals such as RDV and NTV/r were prioritized as reference comparators due to their more consistent use, well-established regulatory status, and robust reporting volume. The utilization of a disproportionality analysis enabled the identification of potential safety signals, through the comparison of the reporting frequency of an adverse reaction for a specific medication in relation to another antiviral [48]. The *ROR* was calculated using a standard 2 × 2 contingency table, with the following formula [49]:

$$ROR=\frac{a/c}{b/d}=\frac{a\times d}{b\times c}$$

where *a*—number of reports of the specific adverse event for the antiviral of interest; *b*—other events for the same antiviral; *c*—the same event reported for comparator antivirals; and *d*—other events for the comparator drugs.

Confidence intervals (95%) for the ROR were calculated using the logarithmic standard error (*SE*) method, according to the following formula [49]:

$$SE[\ln\left(ROR\right)]=\sqrt{\frac{1}{a}+\frac{1}{b}+\frac{1}{c}+\frac{1}{d}\;}$$

$${CI}_{95\%}=\exp(\ln(ROR)\pm 1.96\times SE[\ln\left(ROR)\right])$$

According to EMA methodology, a signal is considered disproportionate if there are at least 5 reported cases, and the lower limit of the 95% CI is greater than 1, indicating significantly more frequent reporting of that adverse reaction for the antiviral under analysis [50].

Disproportionality analysis using ROR has recognized value in signal detection [51] but is subject to important methodological limitations. As it relies exclusively on reported cases, it does not reflect true incidence rates and cannot account for the actual number of individuals exposed to each drug. Moreover, ROR are not adjusted for potential confounders such as patient comorbidities, concomitant treatments, or disease severity. The method is also highly sensitive to reporting biases, which may be influenced by media coverage or regulatory attention. Although a lower 95% confidence interval above 1 is commonly used to indicate statistical significance, such signals are not conclusive of causality and must be interpreted within the broader clinical and pharmacological context. Finally, testing across multiple drug-event combinations without correction increases the likelihood of false-positive findings.

## 3. Results

### 3.1. Distribution of Reported Cases Across Antivirals

Data extracted from the EudraVigilance database revealed notable differences in the reporting frequency of adverse events among the antivirals evaluated for COVID-19. Among the approved treatments, NTV/r accounted for the highest share of reports (13.42%), followed by RDV with 6.34%, reflecting their respective use in outpatient and inpatient settings. FVP, although used off-label in certain countries, generated a limited number of reports (0.24%), likely due to its restricted use and potential underreporting.

**Figure 1:**
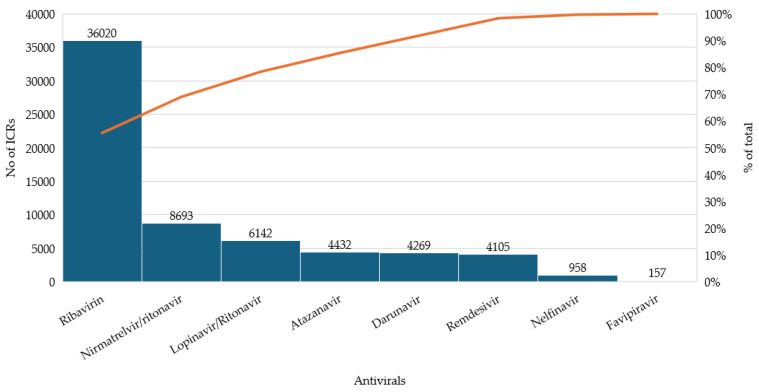
Distribution of individual case safety reports (ICSRs) for selected antivirals.

The largest proportion of reports (>80%) was associated with repurposed antivirals that were not officially adopted in COVID-19 treatment guidelines. RBV alone contributed 55.61% of all cases, while LPV/r, ATV, DRV, and NFV also presented considerable proportions. These differences are visually summarized in Figure 1, highlighting the relative impact of each antiviral within the overall dataset.

The analysis of adverse events per case revealed that RDV had the lowest ADR-to-ICSR ratio (1.64). NTV/r and FVP showed moderately higher ratios, with 2.30 and 2.10 ADRs per case, respectively. In contrast, repurposed antivirals such as NFV (2.69), LPV/r (2.47), and RBV (2.34) were associated with higher reaction densities. ATV (2.05) and DRV (2.12) also showed elevated values (Table 2).

### 3.2. Demographic and Reporting Characteristics of Adverse Event Cases

The analysis of ICSRs by age and sex also revealed relevant differences between antivirals (Table 3). Most reactions were reported in adults aged 18–64 years, representing over 60% of cases for all antivirals, except for NTV/r, where the proportion was lower (37.6%), reflecting wider use among older patients. For RDV, the distribution was balanced between adults aged 18–64 and 65–85, each group contributing roughly one-third of reports. Notably, over 10% of RDV-related cases involved patients aged > 85 years, the highest proportion in this age group across all antivirals. Regarding sex distribution, NTV/r was more frequently associated with reports from female patients (61.7%), while other antivirals showed either a more balanced distribution or a slight predominance in males. A higher number of cases with unspecified age or sex was noted for RBV and LPV/r, likely due to reporting limitations rather than usage patterns.

Complementing these demographic characteristics, the distribution of adverse event reports by geographic origin and reporter types also showed distinct patterns among the studied antivirals (Table 4). Most cases originated from outside the European Economic Area (non-EEA) for all drugs, except for ATV and DRV, where more than half of the reports came from within the EEA. RDV was associated with 64.1% of reports from non-EEA sources, while NTV/r had a nearly even split between EEA (48.4%) and non-EEA (51.6%) regions. In terms of reporter type, most cases across all antivirals were submitted by healthcare professionals, indicating a high level of medical oversight in drug administration. However, NTV/r stood out with a significantly higher proportion of non-healthcare professionals reports (45.3%), likely reflecting its wider outpatient use and direct access by patients.

### 3.3. Severity and System Organ Class (SOC) Distribution of Adverse Events

The analysis of adverse event clinical significance highlighted clear differences among the antivirals studied, as can be seen in Table 5. RDV was associated with a high proportion of serious adverse events (84%), while NTV/r showed a more balanced distribution, with 40.2% of reports classified as non-serious. A similar profile to RDV was observed for several repurposed antivirals, including RBV, NFV, ATV, and LPV/r, where over 85–95% of the reported events were serious.

In terms of the affected system organ classes (SOC), adverse events were concentrated in several categories (Table 6). The most frequently reported adverse events were general disorders and administration site conditions (31.6%), which include non-specific symptoms such as fatigue, fever, and injection site reactions. Other frequent categories were investigations (20.4%), reflecting abnormal laboratory findings (e.g., elevated liver enzymes, altered coagulation), gastrointestinal disorders (19%), and nervous system disorders (17.7%), including headache, dizziness, and confusion. Other frequently reported SOCs included skin and subcutaneous tissue disorders (13.4%), infections and infestations (16.9%), as well as hepatobiliary, renal, psychiatric, and respiratory disorders, each representing between 5 and 10% of total reports. Less frequently represented were endocrine, congenital, and reproductive system disorders, each accounting for under 2% of cases.

SOC distribution patterns varied across antivirals. NTV/r was predominantly associated with neurological (39.8%) and gastrointestinal (37.8%) events. RDV had most reports in general (27.1%) and investigation-related SOCs (26.1%), followed by hepatobiliary (10.5%) and renal disorders (10.5%). FVP, with fewer total reports (*n* = 157), showed no dominant pattern but included general (19.7%), gastrointestinal (17.8%), and nervous system disorders (7.6%). RBV, the most reported antiviral, showed high counts across nearly all SOCs, reflecting its broad systemic impact. LPV/r and ATV had high proportions in gastrointestinal, general, and hepatobiliary SOCs, while DRV showed a more even distribution with peaks, in general, and nervous system disorders. Despite its low volume, NFV was notably represented in general (28.8%) and gastrointestinal (21.3%) categories.

### 3.4. Disproportionality Analysis of Reporting Odds Ratios Highlighting Comparative Safety Signals

The disproportionality analysis (ROR) provided further insight into the safety profiles of RDV and NTV/r in comparison with repurposed antivirals. All SOC categories with five or more reports were included in the analysis, and the most relevant signals are summarized in Table 7.

**Figure 2:**
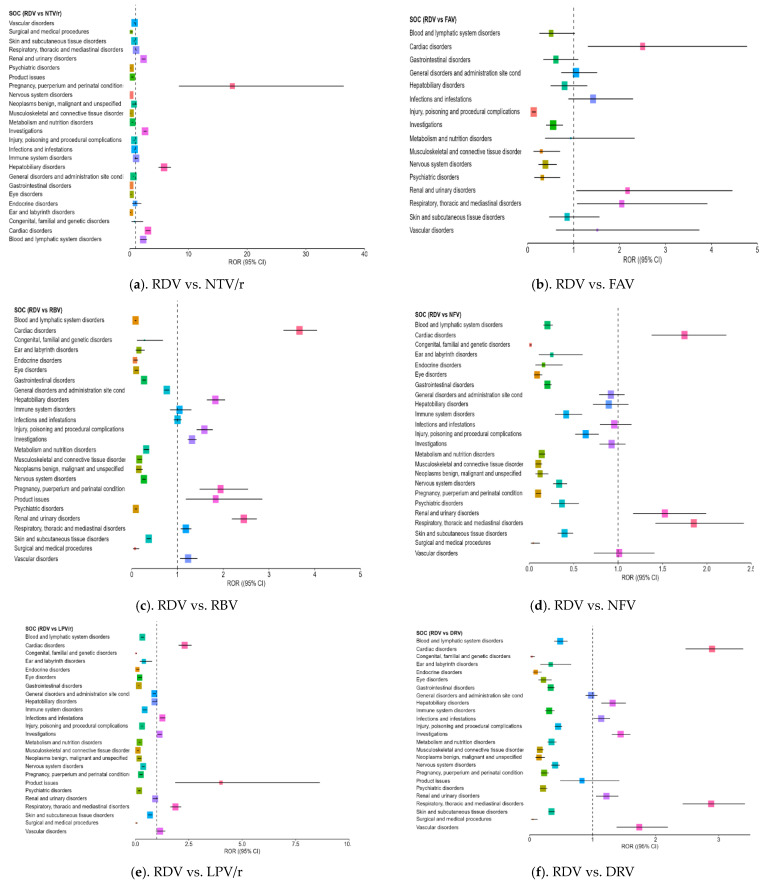

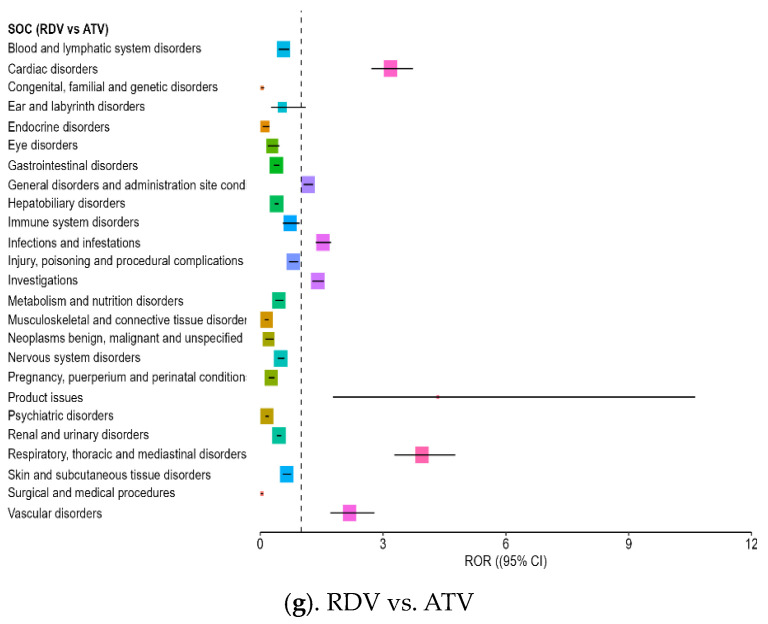
Forest plot illustrating the RORs for adverse events associated with remdesivir compared to other antivirals. Each point along the *X*-axis represents the estimated ROR for a specific SOC, with horizontal bars indicating the 95% CI. The vertical dotted line marks the neutral value of ROR = 1, which serves as the reference threshold. Points located to the right of this line suggest a higher frequency of reports for RDV, while those to the left indicate more frequent reporting for the comparator drug. RDV—remdesivir; NTV/r—nirmatrelvir/ritonavir; FAV—favipiravir; LPV/r—lopinavir/ritonavir; RBV—ribavirin; NFV—nelfinavir; ATV—atazanavir; DRV—darunavir.

RDV showed a higher number of positive ROR signals (ROR > 1) than any other antiviral, particularly in clinically important SOC categories (Figure 2a–g). The most consistent and clinically relevant findings were observed in the following:

– Cardiac disorders: Strong ROR signals were found across comparisons, including ROR = 3.18 vs. ATV (CI: 2.71–3.73) and ROR = 3.10 vs. NTV/r (CI: 2.732–3.531), suggesting possible cardiotoxicity in some patients.
– Hepatobiliary disorders: RDV was associated with a significantly higher reporting rate compared to NTV/r (ROR = 5.85, CI: 4.88–7.01) and ROR > 1 against RBV and DRV, supporting its known hepatic impact.
– Renal and urinary disorders: Elevated RORs (>2) were observed across several comparisons, indicating frequent reports of renal impairment or dysfunction, aligning with the need for renal monitoring in clinical use.
– Investigations (abnormal lab findings): Disproportionate reporting may reflect increased clinical monitoring in hospitalized patients receiving RDV, rather than direct systemic effects of the drug,
– General disorders and administration site conditions: RDV also showed an excess of reporting in this broad category, which includes symptoms such as fatigue, fever, or injection site reactions (ROR = 1.73 vs. NTV/r).

Additional ROR > 1 signals were noted in vascular disorders, product issue, and pregnancy, puerperium and perinatal conditions outcomes (e.g., spontaneous abortion, preterm labor), mostly in comparisons with repurposed antivirals like ATV, DRV, LPV/r, and RBV.

**Figure 3:**
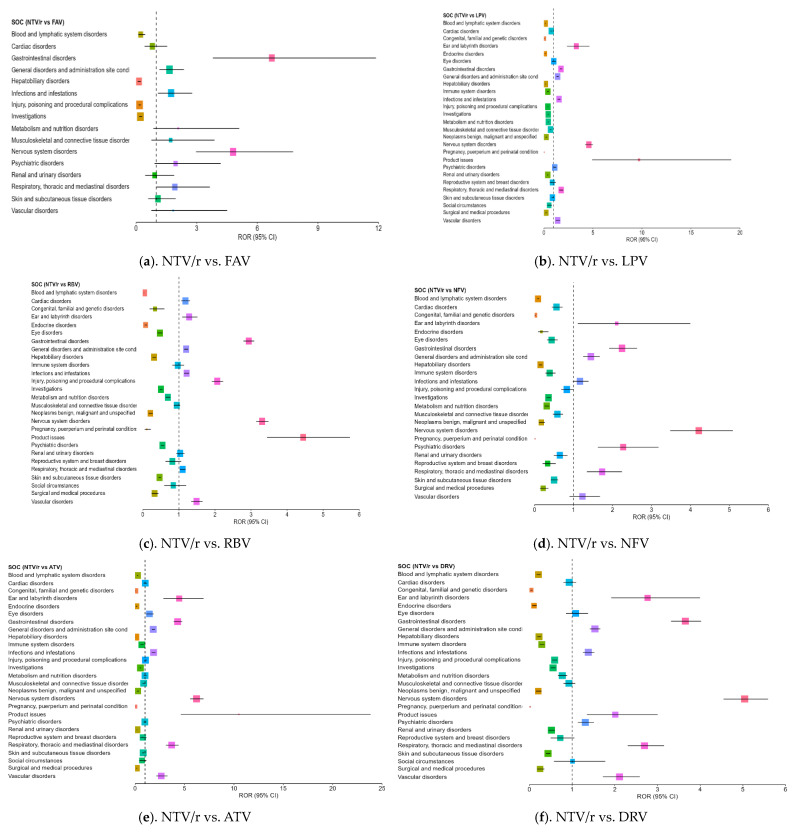
Forest plot illustrating the RORs for adverse events associated with NTV/r, compared to other antivirals.

While NTV/r generally presented a milder safety profile, a few specific SOC categories exhibited disproportionate reporting, when compared with other antivirals (Figure 3a–f):

– Psychiatric disorders: Reports were notably higher than for some comparators, including ROR = 2.17 vs. NFV (CI: 1.62–3.18), indicating the need for further clinical observation in this area.
– Product issues: NTV/r was significantly associated with technical or quality-related complaints (ROR = 1.80 vs. RDV; CI: 1.18–2.76), possibly due to its widespread use in outpatient settings.
– Vascular disorders: Mildly elevated RORs (1.40–2.64) were observed in some comparisons, warranting further exploration in vulnerable populations.

Additional ROR > 1 signals were noted in ear and labyrinth disorders, eye disorders, infections and infestations, nervous system, disorders and respiratory, thoracic and mediastinal disorders. Although the overall safety profile of NTV/r appears more favorable, especially in systemic reactions, these signals suggest a need for targeted monitoring, particularly in patients with psychiatric history.

## 4. Discussion

Antiviral therapy in COVID-19 has evolved rapidly throughout the pandemic, as part of an ongoing effort to control viral replication and prevent progression to severe forms of the disease. The virus and its variations are dynamic, necessitating continual evaluation of therapy efficacy and quick adaptation of therapeutic methods [3,52]. In this diverse and constantly evolving therapeutic context, analyzing the safety profile of antivirals, whether officially authorized or repurposed, becomes essential for optimizing clinical decision-making. Pharmacovigilance results can significantly contribute to understanding the differences between drugs in terms of the frequency, severity, and types of adverse reactions, with direct implications for treatment choices, especially in vulnerable populations.

In our analysis, the comparative safety profiles observed across the antivirals included in this study reflect both their clinical application and regulatory status. NTV/r, primarily used in outpatient care, showed a higher proportion of non-serious reports and a lower average number of adverse reactions per case (2.30), aligning with its use in lower-risk populations. In contrast, RDV, used mainly in hospitalized patients, was associated with a higher proportion of serious cases (84%) and a lower ADR-to-case ratio (1.64), likely reflecting more focused reporting in severe clinical settings. This observed disparity warrants further exploration considering real-world clinical data comparing the safety outcomes of these antivirals in different care settings. A retrospective observational study involving 2140 hospitalized patients treated with remdesivir reported that 66.2% experienced at least one ADR, and 13.8% experienced severe ADRs, such as hepatic injury and anemia [53]. In contrast, a multicenter prospective observational study of 480 nonhospitalized adult patients receiving oral antiviral agents found that 32.9% of those treated with nirmatrelvir/ritonavir experienced adverse events, which were generally mild to moderate and resolved after discontinuation [54]. Taken together, the outcomes show that the higher serious cases rate associated with remdesivir in pharmacovigilance data may be confounded by indication and clinical context. Such limitations are inherent in real-world safety databases, which often lack detailed clinical information needed to adjust for disease severity and comorbid conditions.

Repurposed antivirals such as ribavirin, lopinavir/ritonavir, and nelfinavir accounted for most of the safety reports in the dataset and showed a broader range of reactions. These substances, though not approved specifically for COVID-19, had longstanding use in other indications, and the accumulated clinical exposure may partly explain the volume and diversity of reported adverse events.

The results obtained through disproportionality analysis (ROR), indicating specific clinical signals that warrant continued monitoring. A ROR above 1 was consistently observed in comparisons with repurposed antivirals, indicating a systemic reactive profile that supports the recommendation for close monitoring during administration.

To contextualize these pharmacovigilance signals, Table 8 presents a cross-comparison of SOC-level disproportionality signals with safety information reported in official product characteristics (SmPCs) and existing literature. The analysis revealed several concordant adverse reactions for both RDV and NTV/r, particularly in categories such as hepatobiliary disorders, gastrointestinal disorders, nervous system disorders, and general disorders and administration site conditions, which were consistently reported and documented. However, some discordances were identified. For Remdesivir, unlisted but reported effects included cardiac disorders, infections and infestation, renal and urinary disorders, respiratory disorders, and vascular events. Similarly, for NTV/r, adverse events such as cardiac disorders, ear and labyrinth disorders, eye disorders, infections and infestations, and respiratory and vascular events appeared in post-marketing reports but were not included in the SmPC. These findings may not correspond to specific newly identified adverse reactions but rather indicate areas of increased reporting that warrant further clinical monitoring and investigation.

These findings highlight the essential role of post-marketing surveillance in detecting safety signals that may not emerge during pre-authorization clinical trials, particularly in the context of emergency approvals or accelerated development. When signals identified through spontaneous reporting align with those observed in published real-world studies, their relevance is reinforced, and the understanding of potential drug-related risks becomes more robust. To ensure that clinical guidance remains up to date and reflective of real-world use, it is important for regulatory authorities and manufacturers to regularly reassess and revise SmPCs based on emerging safety data. Doing so helps to strengthen pharmacovigilance systems and supports safer, evidence-informed decision-making in everyday clinical practice.

While the ROR analysis for repurposed antivirals (e.g., LPV/r, DRV, RBV) generally showed lower or neutral values compared to RDV and NTV/r, several SOC-level signals emerged that may reflect cumulative exposure, underlying vulnerabilities, or complex treatment regimens. For example, LPV/r presented elevated signals in metabolism and procedural complications, and Ribavirin was associated with psychiatric disorders, consistent with previous literature linking it to mood disturbances, particularly in patients with pre-existing psychiatric conditions [67,86]. Although many adverse events reported for these agents are well-known from prior indications, these findings underscore the need for continued monitoring, especially in polypharmacy settings or vulnerable populations. However, before interpreting these results, several methodological considerations must be acknowledged. This study involved multiple comparisons across a wide range of SOCs and antiviral agents, which inherently increases the risk of false-positive findings. Although formal adjustments for multiple testing (e.g., Bonferroni correction or false discovery rate control) were not applied, consistent with standard practice in signal detection, this should be considered when evaluating the results [87,88]. The findings should therefore be interpreted as exploratory and hypothesis-generating rather than confirmatory. While the threshold of a lower 95% confidence interval (CI) above 1 is widely used to identify statistically significant disproportionality, this does not necessarily indicate clinical relevance. The magnitude of ROR values should be considered alongside the severity and plausibility of the reported adverse events, recognizing that small but statistically significant signals may still warrant closer scrutiny [51]. Reporting in spontaneous systems is voluntary and influenced by awareness or media coverage, which may result in bias [88,89]. Causality cannot be established from these data, and confounding factors such as comorbidities or co-medications may influence results [90]. Also, the choice of reference drug in disproportionality analyses can significantly influence ROR estimates. Differences in clinical use, population characteristics, and drug authorization status limit direct comparability between approved and repurposed antivirals. Additionally, the study did not explore temporal variations in reporting throughout the pandemic period [87]. Future work incorporating a temporal dimension could help clarify how shifts in clinical practice influenced observed safety trends.

Despite these limitations, disproportionality analysis remains a widely accepted method for identifying early safety signals, especially for rare or unexpected events [51]. Similar pharmacovigilance methodologies have been used across therapeutic areas, supporting their relevance beyond antiviral therapy. Comparative studies using WHO-VigiBase or other platforms have successfully identified important signals, such as agranulocytosis with antithyroid agents [91] or myocarditis linked to immune checkpoint inhibitors [92], demonstrating the utility of cross-drug disproportionality analysis for early signal detection and regulatory prioritization. Overall, our findings contribute to a broader understanding of antiviral safety during the COVID-19 pandemic and highlight the importance of adaptive pharmacovigilance systems capable of integrating new evidence and guiding responsible clinical use.

## 5. Conclusions

The present comparative analysis highlights differences between the antivirals used in the treatment of COVID-19 in terms of the volume of adverse reactions, their severity, and the profile of the affected systems. RDV and NTV/r, the antivirals specifically approved for COVID-19, show distinct patterns of reactions, correlated with their routes of administration and target population. RDV is more frequently associated with serious reactions, particularly hepatic and renal, while NTV/r has a profile dominated by digestive and neurological reactions, reflecting its widespread use in outpatient settings.

Repurposed antivirals, such as ribavirin or LPV/r, continue to generate a significant number of reports, largely due to their historical use in other indications. Although most of the reported reactions were serious, their systemic distribution varies, providing useful insights for monitoring the risks associated with each molecule.

This analysis underlines the need for ongoing and differentiated pharmacovigilance monitoring tailored to the profile of each antiviral and provide in order to provide a solid informational basis for optimizing clinical use and risk assessment in antiviral therapies.

## Figures and Tables

**Table 1:** Investigated antiviral drugs, used in the treatment of COVID-19.

Drugs Name/ COVID-19 Approval Status	Original Indication	Indication (COVID-19 Use)	Timeframe and Deployment	Known Adverse Effects	Ref
Remdesivir/ Approved	Developed to treat hepatitis C, investigated for Ebola virus disease and Marburg virus infection [18,19]	Hospitalized patients with moderate-to-severe COVID-19	EMA Conditional Approval—July 2020; widely used globally	Elevated liver enzymes, renal impairment, nausea	[20,21]
Nirmatrelvir and ritonavir/ Approved	Treatment and post-exposure prophylaxis of COVID-19 [22]	Early treatment in non-hospitalized high-risk patients	EMA Conditional Approval—January 2022; moderate use in EU	Dysgeusia, diarrhea, drug interactions, taste disturbances	[23,24]
Favipiravir/ Off-label	Treatment of Influenzas virus infection in Japan [25]	Investigational/off-label use for mild to moderate cases	Used in Japan, India, Russia; not approved by EMA	Hyperuricemia, liver enzyme elevation, teratogenicity	[25,26]
Lopinavir and Ritonavir/ Off-label	Treatment of HIV-1 infection [27]	Off-label use during early pandemic phase	Deployed globally in early 2020; later disfavored due to inefficacy	Diarrhea, QT prolongation, hepatotoxicity	[28,29]
Ribavirin/ Off-label	Treatment of chronic hepatitis C with viral hemorrhagic fever [30]	Investigational use based on past SARS-CoV/MERS data	Limited use, mainly in Asia and MENA during early pandemic	Hemolytic anemia, fatigue, teratogenicity	[31,32]
Nelfinavir/ Off-label	Treatment of HIV-1 infection [33]	Investigational use based on in vitro activity against SARS-CoV-2	Limited use; evaluated in clinical trials during early pandemic	Gastrointestinal intolerance, diarrhea, flatulence	[34,35]
Atazanavir/ Off-label	Treatment of HIV-1 infection [36]	Investigational use; evaluated for SARS-CoV-2 protease inhibition	Limited use; studied in vitro and in clinical settings	Hyperbilirubinemia, jaundice, gastrointestinal disturbances, potential cardiac arrhythmias	[37,38]
Darunavir/ Off-label	Treatment of HIV-1 infection [39]	Investigational use; assessed for potential anti-SARS-CoV-2 activity	Limited use: clinical trials did not demonstrate efficacy	Rash, diarrhea, headache, abdominal pain, potential liver enzyme elevations	[40,41]

COVID-19—coronavirus disease 2019; EMA—European Medicines Agency; EU—European Union; HIV—human immunodeficiency virus; SARS-CoV-2—severe acute respiratory syndrome coronavirus 2; SARS—severe acute respiratory syndrome; MERS—Middle East respiratory syndrome; QT—QT interval; MENA—Middle East and North Africa.

**Table 2:** Average number of reported adverse events per individual case safety report (ICSR) for each antiviral.

Antiviral	Total ADRs	ICSRs	ADRs per ICSR
RDV	6748	4105	1.64
NTV/r	19,977	8693	2.30
FVP	329	157	2.10
LPVr	15,194	6142	2.47
RBV	84,326	36,020	2.34
NFV	2580	958	2.69
ATV	9073	4432	2.05
DRV	9043	4269	2.11

ICSR—individual case safety report; ADR—adverse drug reaction; RDV—remdesivir; NTV/r—nirmatrelvir and ritonavir; FVP—favipiravir; LPVr—lopinavir and ritonavir; RBV—ribavirin; NFV—nelfinavir; ATV—atazanavir; DRV—darunavir.

**Table 3:** Distribution of reported cases by age group and sex for the antivirals analyzed.

Category	RDV	NTV/r	FAV	LPV/r	RBV	NFV	ATV	DRV
Age group								
Not specified	594 (14.5)	1401 (16.1)	24 (14.74)	1399 (22.8)	8049 (22.3)	205 (21.4)	1115 (25.2)	1221 (28.6)
0–1 month	5 (0.1)	5 (0.1)	0 (0.0)	142 (2.3)	36 (0.1)	49 (5.1)	38 (0.9)	72 (1.7)
2 months–2 years	24 (0.6)	1 (0.0)	0 (0.0)	82 (1.3)	48 (0.1)	14 (1.5)	15 (0.3)	13 (0.3)
3–11 years	28 (0.7)	0 (0.0)	0 (0.0)	70 (1.1)	60 (0.2)	21 (2.2)	8 (0.2)	14 (0.3)
12–17 years	28 (0.7)	19 (0.2)	0 (0.0)	87 (1.4)	78 (0.2)	18 (1.9)	36 (0.8)	47 (1.1)
18–64 years	1523 (37.1)	3269 (37.6)	92 (58.97)	3754 (61.1)	22,916 (63.6)	628 (65.6)	2998 (67.6)	2670 (62.5)
65–85 years	1477 (36.0)	3330 (38.3)	39 (25.0)	566 (9.2)	4777 (13.3)	23 (2.4)	214 (4.8)	223 (5.2)
>85 years	426 (10.4)	668 (7.7)	2 (1.28)	42 (0.7)	56 (0.2)	0 (0.0)	8 (0.2)	9 (0.2)
Sex								
Female	1522 (37.1)	5364 (61.7)	60 (37.82)	2210 (36.0)	14,538 (40.4)	324 (33.8)	1393 (31.4)	1384 (32.4)
Male	2406 (58.6)	2956 (34.0)	93 (59.62)	3309 (53.9)	19,064 (52.9)	571 (59.6)	2639 (59.5)	2482 (58.1)
Not specified	177 (4.3)	373 (4.3)	4 (2.56)	630 (10.1)	2418 (6.7)	63 (6.6)	400 (9.0)	404 (9.5)

RDV—remdesivir; NTV/r—nirmatrelvir/ritonavir; FAV—favipiravir; LPV/r—lopinavir/ritonavir; RBV—ribavirin; NFV—nelfinavir; ATV—atazanavir; DRV—darunavir.

**Table 4:** Geographic and reported characteristics.

Category	RDV	NTV/r	FAV	LPV/r	RBV	NFV	ATV	DRV
Geographic origin								
EEA	1472 (35.9)	4206 (48.4)	75 (48.08)	2965 (48.3)	12,582 (34.9)	454 (47.4)	2486 (56.1)	2530 (59.3%)
Non-EEA	2633 (64.1)	4487 (51.6)	82 (51.92)	3177 (51.7)	23,438 (65.1)	504 (52.6)	1946 (43.9)	1739 (40.7%)
Reported group								
Healthcare professional	3829 (93.3)	4753 (54.7)	143 (91.03)	5269 (85.8)	31,712 (88.0)	859 (89.7)	4143 (93.5)	3890 (91.1%)
Non-healthcare professional	276 (6.7)	3940 (45.3)	14 (8.97)	441 (7.2)	4209 (11.7)	51 (5.3)	251 (5.7)	379 (8.9%)
Not specified	0 (0.0)	0 (0.0)	0 (0.0)	432 (7.0)	99 (0.3)	48 (5.0)	38 (0.9)	0 (0.0%)
Total	4105	8693	157	6142	36,020	958	4432	4269

EEA—European Economic Area; Non-EEA—countries outside the European Economic Area; RDV—remdesivir; NTV/r—nirmatrelvir/ritonavir; FAV—favipiravir; LPV/r—lopinavir/ritonavir; RBV—ribavirin; NFV—nelfinavir; ATV—atazanavir; DRV—darunavir.

**Table 5:** Reported serious vs. non-serious cases for each antiviral in numbers (%).

Antiviral	S Cases	NS-Cases	S/NS Ratio
Remdesivir	3448 (84)	657 (16)	5.24
Nirmatrelvir/ritonavir	5202 (59.8)	3491 (40.2)	1.49
Favipiravir	127 (81)	30 (19)	4.23
Lopinavir/ritonavir	5353 (87.2)	789 (12.8)	6.78
Ribavirin	33,442 (92.9)	2578 (7.1)	12.97
Nelfinavir	917 (95.7)	41 (4.3)	22.36
Atazanavir	4066 (91.7)	366 (8.3)	11.11
Darunavir	3565 (83.5)	704 (16.5)	5.06
Total	56,120 (86.6)	8656 (13.4)	6.48

S—serious; NS—non-serious.

**Table 6:** Distribution of ICRs by SOC for selected antivirals used or tested in COVID-19 treatment in number (% *).

SOC Category (Specific Adverse Conditions Most Frequently Reported)	RDV	NTV/r	FVP	LPVr	RBV	NFV	ATV	DRV	Total
General disorders and administration site conditions/ (fatigue, pyrexia, injection site reaction)	1113 (5.4)	3207 (15.6)	41 (0.2)	1812 (8.8)	11,796 (57.6)	276 (1.3)	1070 (5.2)	1178 (5.7)	20,493 (31.6)
Investigations (elevated liver enzymes, abnormal blood glucose, prolonged prothrombin time)	1070 (8.1)	1036 (7.8)	61 (0.5)	1455 (11.0)	7606 (57.5)	264 (2.0)	889 (6.7)	838 (6.3)	13,219 (20.4)
Gastrointestinal disorders (diarrhea, nausea, vomiting, abdominal pain)	216 (1.7)	3282 (26.0)	13 (0.1)	1590 (12.6)	6165 (48.8)	204 (1.6)	547 (4.3)	608 (4.8)	12,625 (19.5)
Nervous system disorders (headache, dizziness, dysgeusia, neuropathy)	207 (1.8)	3460 (30.1)	19 (0.2)	774 (6.7)	6000 (52.1)	130 (1.1)	426 (3.7)	495 (4.3)	11,511 (17.8)
Blood and lymphatic system disorders (anemia, neutropenia, thrombocytopenia)	124 (1.1)	117 (1.0)	9 (0.1)	534 (4.7)	9910 (87.6)	126 (1.1)	231 (2.0)	258 (2.3)	11,309 (17.5)
Infections and infestations (COVID-19, pneumonia, sepsis, oral candidiasis)	707 (6.5)	1755 (16.1)	20 (0.2)	868 (7.9)	6207 (56.8)	171 (1.6)	530 (4.9)	661 (6.1)	10,919 (16.9)
Skin and subcutaneous tissue disorders (rash, pruritus, urticaria)	272 (3.1)	715 (8.2)	12 (0.1)	576 (6.6)	5788 (66.7)	145 (1.7)	439 (5.1)	727 (8.4)	8674 (13.4)
Psychiatric disorders (insomnia, anxiety, confusional state, depression)	60 (0.8)	727 (10.3)	7 (0.1)	468 (6.6)	5133 (72.4)	37 (0.5)	377 (5.3)	278 (3.9)	7087 (10.9)
Injury, poisoning and procedural complications (infusion site extravasation, overdose, administration error)	400 (5.8)	1072 (15.5)	71 (1.0)	1573 (22.8)	2295 (33.3)	139 (2.0)	524 (7.6)	822 (11.9)	6896 (10.6)
Respiratory, thoracic and mediastinal disorders (dyspnea, respiratory failure, cough)	502 (8.2)	1003 (16.3)	10 (0.2)	425 (6.9)	3801 (61.7)	67 (1.1)	151 (2.5)	197 (3.2)	6156 (9.5)
Metabolism and nutrition disorders (hyperglycemia, electrolyte imbalance, decreased appetite)	123 (2.2)	558 (10.1)	5 (0.1)	828 (14.9)	3228 (58.2)	173 (3.1)	282 (5.1)	349 (6.3)	5546 (8.6)
Hepatobiliary disorders (hepatic enzyme increased, hepatitis, liver injury)	431 (8.7)	171 (3.4)	20 (0.4)	710 (14.3)	2172 (43.7)	111 (2.2)	1006 (20.2)	349 (7.0)	4970 (7.7)
Renal and urinary disorders (acute kidney injury, increased creatinine, renal failure)	429 (9.5)	409 (9.1)	8 (0.2)	687 (15.2)	1640 (36.3)	68 (1.5)	902 (20.0)	373 (8.3)	4516 (7.0)
Musculoskeletal and connective tissue disorders (myalgia, arthralgia, back pain)	48 (1.1)	553 (12.8)	6 (0.1)	563 (13.1)	2420 (56.2)	100 (2.3)	323 (7.5)	292 (6.8)	4305 (6.6)
Cardiac disorders (bradycardia, tachycardia, cardiac arrest, palpitations)	597 (16.5)	452 (12.5)	10 (0.3)	422 (11.6)	1598 (44.1)	85 (2.3)	225 (6.2)	237 (6.5)	3626 (5.6)
Vascular disorders (hypertension, hypotension, deep vein thrombosis)	195 (7.5)	496 (19.0)	5 (0.2)	253 (9.7)	1403 (53.7)	45 (1.7)	99 (3.8)	119 (4.6)	2615 (4.0)
Eye disorders (visual impairment, blurred vision, retinal changes)	22 (0.9)	225 (8.8)	2 (0.1)	158 (6.2)	1916 (74.8)	55 (2.1)	80 (3.1)	102 (4.0)	2560 (4.0)
Immune system disorders (hypersensitivity, anaphylactic reaction, cytokine release syndrome)	88 (5.0)	174 (9.9)	1 (0.1)	293 (16.7)	741 (42.2)	48 (2.7)	129 (7.4)	281 (16.0)	1755 (2.7)
Neoplasms benign, malignant and unspecified-including cysts and polyps (lymphoma, leukemia, benign neoplasm)	19 (1.2)	56 (3.6)	1 (0.1)	161 (10.2)	1071 (68.1)	35 (2.2)	101 (6.4)	129 (8.2)	1573 (2.4)
Endocrine disorders (thyroid dysfunction, diabetes mellitus, adrenal insufficiency)	9 (0.6)	21 (1.4)	2 (0.1)	134 (9.2)	1096 (75.4)	13 (0.9)	84 (5.8)	95 (6.5)	1454 (2.2)
Pregnancy, puerperium and perinatal conditions (spontaneous abortion, preterm labor, low birth weight)	65 (4.7)	8 (0.6)	3 (0.2)	358 (25.7)	296 (21.2)	133 (9.5)	253 (18.1)	278 (19.9)	1394 (2.2)
Surgical and medical procedures (catheter placement, device removal, surgical intervention)	5 (0.4)	51 (4.4)	0 (0.0)	166 (14.4)	637 (55.2)	25 (2.2)	172 (14.9)	98 (8.5)	1154 (1.8)
Ear and labyrinth disorders (tinnitus, hearing loss, vertigo)	11 (1.2)	189 (20.6)	1 (0.1)	41 (4.5)	611 (66.5)	10 (1.1)	22 (2.4)	34 (3.7)	919 (1.4)
Congenital, familial and genetic disorders (congenital anomaly, genetic mutation, familial disorder)	5 (0.6)	13 (1.7)	0 (0.0)	231 (29.4)	157 (20.0)	95 (12.1)	134 (17.0)	151 (19.2)	786 (1.2)
Reproductive system and breast disorders (menstrual disorder, gynecomastia, erectile dysfunction)	2 (0.3)	68 (11.7)	2 (0.3)	56 (9.6)	343 (58.8)	22 (3.8)	44 (7.5)	46 (7.9)	583 (0.9)
Social circumstances (social isolation, caregiver burden, living conditions affected)	4 (1.3)	37 (11.6)	0 (0.0)	49 (15.4)	181 (56.7)	3 (0.9)	27 (8.5)	18 (5.6)	319 (0.5)
Product issues (packaging error, product contamination, device malfunction)	24 (7.8)	122 (39.9)	0 (0.0)	9 (2.9)	115 (37.6)	0 (0.0)	6 (2.0)	30 (9.8)	306 (0.5)

* Percentages shown in the antiviral-specific columns represent the proportion of adverse reactions attributed to each antiviral within the total number of reports for that specific SOC category. Percentages in the Total column reflect the share of each SOC in relation to the total number ICSRs included in the dataset (N = 64,776). RDV—remdesivir; NTV/r—nirmatrelvir/ritonavir; LPV/r—lopinavir/ritonavir; RBV—ribavirin; NFV—nelfinavir; ATV—atazanavir; DRV—darunavir; FVP—favipiravir.

**Table 7:** Significant disproportionality signals for RDV and NTV/r versus other antivirals, by SOC category.

SOC	Comp	ROR RDV	CI Low RDV	CI High RDV	ROR NTV/r	CI Low NTV/r	CI High NTV/r
Cardiac disorders	ATV	3.182	2.712	3.733	-	-	-
	DRV	2.895	2.474	3.388	-	-	-
	FVP	2.502	1.311	4.775	-	-	-
	LPV/r	2.307	2.022	2.631	-	-	-
	NFV	1.748	1.376	2.22	-	-	-
	RBV	3.666	3.316	4.052	1.181	1.061	1.315
Ear and labyrinth disorders	ATV	-	-	-	4.455	2.861	6.938
	DRV	-	-	-	2.768	1.918	3.996
	LPV/r	-	-	-	3.307	2.356	4.643
	NFV	-	-	-	2.107	1.111	3.994
	RBV	-	-	-	1.288	1.092	1.519
Eye disorders	ATV	-	-	-	1.445	1.117	1.87
Gastrointestinal disorders	ATV	-	-	-	4.308	3.9	4.758
	DRV	-	-	-	3.652	3.317	4.021
	FVP	-	-	-	6.719	3.802	11.872
	LPV/r	-	-	-	1.736	1.616	1.866
	NFV	-	-	-	2.242	1.909	2.633
	RBV	-	-	-	2.937	2.79	3.092
General disorders and administration site conditions	ATV	1.169	1.06	1.288	1.837	1.693	1.993
	DRV	-	-	-	1.534	1.416	1.662
	FVP	-	-	-	1.654	1.155	2.368
	LPV/r	-	-	-	1.397	1.302	1.498
	NFV	-	-	-	1.445	1.248	1.672
	RBV	-	-	-	1.2	1.143	1.261
Hepatobiliary disorders	DRV	1.318	1.136	1.528	-	-	-
	RBV	1.828	1.64	2.038	-	-	-
Infections and infestations	ATV	1.532	1.356	1.73	1.862	1.677	2.068
	DRV	1.136	1.011	1.275	1.381	1.252	1.523
	FVP	-	-	-	1.733	1.081	2.778
	LPV/r	1.264	1.134	1.409	1.537	1.406	1.68
	RBV	-	-	-	1.215	1.145	1.289
Injury, poisoning and procedural complications	RBV	1.587	1.419	1.774	2.067	1.915	2.232
Investigations	ATV	1.405	1.27	1.555	-	-	-
	DRV	1.443	1.302	1.6	-	-	-
	LPV/r	1.136	1.037	1.244	-	-	-
	RBV	1.317	1.223	1.418	-	-	-
Nervous system disorders	ATV	-	-	-	6.218	5.577	6.932
	DRV	-	-	-	5.041	4.547	5.588
	FVP	-	-	-	4.802	2.967	7.773
	LPV/r	-	-	-	4.586	4.205	5.001
	NFV	-	-	-	4.211	3.483	5.092
	RBV	-	-	-	3.308	3.143	3.482
Pregnancy, puerperium and perinatal conditions	RBV	1.942	1.482	2.545	-	-	-
Product issues	ATV	4.338	1.771	10.624	10.5	4.623	23.85
	DRV				2.011	1.347	3.004
	LPV/r	4.008	1.861	8.63	9.7	4.925	19.104
	RBV	1.836	1.181	2.854	4.444	3.441	5.74
Psychiatric disorders	DRV	-	-	-	1.31	1.135	1.512
	NFV	-	-	-	2.272	1.621	3.183
Renal and urinary disorders	DRV	1.219	1.053	1.41	-	-	-
	FVP	2.174	1.06	4.458	-	-	-
	NFV	1.527	1.171	1.993	-	-	-
	RBV	2.446	2.188	2.735	-	-	-
Respiratory, thoracic and mediastinal disorders	ATV	3.95	3.276	4.763	3.698	3.104	4.406
	DRV	2.88	2.428	3.416	2.696	2.303	3.156
	FVP	2.048	1.072	3.913	1.917	1.007	3.65
	LPV/r	1.874	1.636	2.147	1.755	1.558	1.975
	NFV	1.853	1.421	2.416	1.735	1.342	2.242
	RBV	1.181	1.069	1.304	1.106	1.027	1.19
Vascular disorders	ATV	2.183	1.707	2.791	2.648	2.128	3.296
	DRV	1.739	1.379	2.194	2.11	1.722	2.587
	LPV/r	-	-	-	1.408	1.206	1.645
	RBV	1.231	1.056	1.435	1.493	1.344	1.659

SOC—system organ class; ROR—reporting odds ratio; - statistically insignificant values; CI—confidence interval, RDV—remdesivir; NTV/r—nirmatrelvir/ritonavir; ATV—atazanavir; DRV—darunavir; FVP—favipiravir; LPV/r—lopinavir/ritonavir; NFV—nelfinavir; RBV—ribavirin.

**Table 8:** Comparative summary of the adverse effects for studied antiviral drugs.

SOC Categories ROR > 1 (EudraVigilance)	Listed in SmPC	Findings in Other Studies	Observations
Remdesivir [55]			
Cardiac disorders RORs (1.75–3.67) vs. all	No (sinus bradycardia * post-marketing)	Bradycardia [14,56,57,58], induced cardiotoxicity [14,59]	Noted in the literature, not consistently reflected in labeling
General disorders and administration site conditions ROR = 1.17 vs. ATV	Yes (infusion-related reaction ^r^)	No data found	Listed in SmPC
Hepatobiliary disorders RORs (1.32–5.85) vs. DRV, RBV, and NTV/r	Yes (↑alanine aminotransferase/aspartate aminotransferase ^vc^)	Elevated hepatic enzymes [60,61]; liver injury [62,63]	Listed in SmPC, consistent with known hepatic effects
Infections and Infestations RORs (1.14–1.53) vs. ATV, DRV, and LPV/r	No	No data found	Not listed in SmPC, disproportionate reporting can be caused by its antiviral mechanism that may have an influence on the immune system, increasing the risk of opportunistic infections
Injury, poisoning and procedural Complications ROR = 1.59 vs. RBV	Yes (infusion-related reaction ^r^)	Injection site reaction [64,65]	Listed in SmPC
Investigations (abnormal lab findings) RORs (1.14–2.61) vs. ATV, DRV, LPV/r, RBV, and NTV/r	Yes (prothrombin time prolonged ^vc^)	Rise in blood sugars [66,67]; low albumin, low potassium, low red blood cell count, low platelet count [68]	Listed in SmPC
Pregnancy and reproductive system disorders RORs (1.94, 17.47) vs. RBV and NTV/r	Precautionary guidance only; no adverse events listed	Limited published evidence; no confirmed safety signal to date [64]	Not listed in SmPC; low case numbers but disproportionate reporting suggests need for targeted monitoring in pregnancy-related contexts
Product issues RORs (1.84–4.34) vs. ATV, LPV/r, and RBV	No	Limited post-marketing reports on formulation and packaging; no peer-reviewed studies available	Not listed in SmPC—may indicate formulation or distribution-related concerns
Renal and urinary disorders RORs (1.22–2.45) vs. DRV, FVP, NFV, RBV and NTV/r	No	Kidney injury [62,69]; kidney disorders [70] acute kidney injury [71]; decreased glomerular filtration rate [67]	Noted in the literature, not currently listed in SmPC- may reflect underrecognized risk.
Respiratory, thoracic and mediastinal disorders RORs (1.07–3.95) vs. all	No	Respiratory failure [67,72,73]	Noted in the literature, not currently listed in SmPC may reflect disease severity rather than a direct effect of the drug
Vascular disorders RORs (1.23–2.18) vs. ATV, DRV, and RBV	No	No data found	Not listed in SmPC, may be related to disease progression, patient comorbidities, or the related cardiac adverse events presented in the literature
Nirmatrelvir/ritonavir [74]			
Cardiac disorders ROR= 1.18 vs. ATV	No	Troponin I level changes [75]; DDIs in patients with cardiac diseases [76]; Cardiovascular sequelae [77]; Bradycardia [78]	Not listed in SmPC; may reflect cardiac risk, drug interactions or may be due to patients’ comorbidities, without confirmed causality
Ear and labyrinth disorders RORs (1.28–4.45) vs. all except FAV	No	No data found	Not listed in SmPC; may be caused by the infection itself which alter cochlear function
Eye disorders ROR= 1.44 vs. ATV	No	Retinal impairment [79]	Not listed in SmPC; may be caused just by ritonavir; ongoing pharmacovigilance and further studies are essential
Gastrointestinal disorders RORs (1.74–6.72) vs. all	Diarrhea ^c^, vomiting ^c^, nausea ^c^ Abdominal pain	Dysgeusia and diarrhea [80]; diarrhea, stomach pain [81]; nausea [54]	Listed in SmPC
General disorders and administration site conditions RORs (1.20–1.81) vs. all	Malaise ^r^	Pale-colored stools, chromaturia, yellow skin, tongue coating [82]	Listed in SmPC
Infections and infestations RORs (1.21–1.84) vs. ATV, DRV, FVP, LPV/r, and RBV	No	No data found	Not listed in SmPC; may reflect opportunistic or secondary infections during COVID-19
Injury, poisoning and procedural complications ROR= 1.27 vs. RBV	No	No data found	Not listed in SmPC; may result from administration errors or unrelated incidents during treatment; no confirmed causal link to the drug itself
Nervous system disorders	Dysgeusia ^c^, headache ^c^	Neurological sequelae [77]; neurological adverse reactions [83]; Headache [84]; dizziness [54]	Listed in SmPC
Product issues RORs (2.01–10.5) vs. ATV, DRV, LPV/r, and RBV	No	Limited post-marketing reports on formulation and packaging; no peer-reviewed studies found.	Not currently listed in SmPC—may indicate formulation or distribution-related concerns.
Psychiatric disorders RORs (1.31, 2.27) vs. DRV and NFV	Yes (Dysgeusia, headache ^c^)	Neuropsychiatric effects [80,82,84]	Listed in SmPC
Respiratory, thoracic and mediastinal disorders RORs (1.11–3.70) vs. all	No	Dyspnea [85]	Not listed in SmPC; may reflect COVID-19 progression rather than a direct effect of the drug; it is a cause for worry, particularly in people who have pre-existing respiratory disorders.
Vascular disorders RORs (1.41–2.65) vs. ATV, DRV, LPV/r, and RBV	No	No data found	Not listed in SmPC; may reflect COVID-19–related vascular risk or drug interactions, or can be linked with the cardiac disorders

SOC—system organ classes; ROR—reporting odds ratio; SmPC—Summary of Product Characteristics; DDIs—drug–drug interactions; NTV/r—nirmatrelvir/ritonavir; FVP—favipiravir; LPV/r—lopinavir/ritonavir; RBV—ribavirin; NFV—nelfinavir; ATV—atazanavir; DRV—darunavir; ^vc^—very common (≥1/10); ^c^—common (≥1/100 to <1/10); ^r^—rare (≥1/10,000 to <1/1000). * Reported in post-marketing, usually normalized within 4 days following last remdesivir administration without additional intervention.

## Data Availability

The data presented in this study were derived from publicly available resources. Specifically, data were obtained from the EudraVigilance (EV) database, maintained by the European Medicines Agency (EMA), accessible at https://www.adrreports.eu (accessed on 12 February 2025). No new datasets were generated or deposited during the study.

## Abbreviations

The following abbreviations are used in this manuscript:

ADRs	Adverse drug reactions
ATV	Atazanavir
COVID-19	Coronavirus disease 2019
DRV	Darunavir
EEA	European Economic Area
EMA	European Medicines Agency
EV	EudraVigilance
FVP	Favipiravir
HIV	Human immunodeficiency virus
LPV/r	Lopinavir/ritonavir
NFV	Nelfinavir
NTV/r	Nirmatrelvir/ritonavir
RDV	Remdesivir
RBV	Ribavirin
RTV	Ritonavir
SARS-CoV-2	Severe acute respiratory syndrome coronavirus 2
SOC	System organ class
WHO	World Health Organization
SmPCs	Official product characteristics
ICSr	Individual case safety reports
